# Histopathologic and MR Imaging Appearance of Spontaneous and Radiation-Induced Necrosis in Uveal Melanomas: Initial Results

**DOI:** 10.3390/cancers14010215

**Published:** 2022-01-02

**Authors:** Pietro Valerio Foti, Corrado Inì, Giuseppe Broggi, Renato Farina, Stefano Palmucci, Corrado Spatola, Rocco Luca Emanuele Liardo, Roberto Milazzotto, Luigi Raffaele, Vincenzo Salamone, Rosario Caltabiano, Lidia Puzzo, Andrea Russo, Michele Reibaldi, Antonio Longo, Paolo Vigneri, Massimo Venturini, Francesco Giurazza, Teresio Avitabile, Antonio Basile

**Affiliations:** 1 Department of Medical Surgical Sciences and Advanced Technologies “G.F. Ingrassia”, University of Catania —Radiology I Unit, University Hospital Policlinico “G. Rodolico-San Marco”, Via Santa Sofia 78, 95123 Catania, Italy; corrado.ini@gmail.com (C.I.); radfaro@hotmail.com (R.F.); spalmucci@sirm.org (S.P.); cor_spatola@hotmail.com (C.S.); Lucaliardo@hotmail.com (R.L.E.L.); Robertomilazz@hotmail.it (R.M.); raffaele@lns.infn.it (L.R.); vincenzosalamone@hotmail.com (V.S.); basile.antonello73@gmail.com (A.B.); 2 Department of Medical Surgical Sciences and Advanced Technologies “G.F. Ingrassia”—Section of Anatomic Pathology, University of Catania, Via Santa Sofia 87, 95123 Catania, Italy; giuseppe.broggi@gmail.com (G.B.); rosario.caltabiano@unict.it (R.C.); lipuzzo@unict.it (L.P.); 3 Department of Ophthalmology, University of Catania, Via Santa Sofia 78, 95123 Catania, Italy; andrearusso2000@hotmail.com (A.R.); mreibaldi@libero.it (M.R.); antlongo@unict.it (A.L.); t.avitabile@unict.it (T.A.); 4 Department of Clinical and Experimental Medicine, Center of Experimental Oncology and Hematology, University Hospital Policlinico “G. Rodolico-San Marco”, Via Santa Sofia 78, 95123 Catania, Italy; vigneripaolo@gmail.com; 5 Diagnostic and Interventional Radiology Department, Circolo Hospital, Insubria University, Viale Luigi Borri 57, 21100 Varese, Italy; massimo.venturini@uninsubria.it; 6 Vascular and Interventional Radiology Department, Cardarelli Hospital, Via A. Cardarelli 9, 80131 Naples, Italy; francescogiurazza@hotmail.it

**Keywords:** eye (A01.456.505.420), uvea (A09.371.894), eye neoplasms (C04.588.364), uveal neoplasms (C04.588.364.978), melanoma (C04.557.465.625.650.510), magnetic resonance imaging (E01.370.350.825.500), necrosis (C23.550.717), radiation injuries (C26.733), radiotherapy (E02.815), proton therapy (E02.815.250.500)

## Abstract

**Simple Summary:**

Uveal melanomas may undergo necrosis, both spontaneously or following radiotherapy. Nowadays radiotherapy is the preferred treatment, whereas enucleation of the eye is used in selected cases. In order to differentiate the effects of radiotherapy from spontaneous degenerative changes in uveal melanomas, we compared the appearance of necrosis, both from a histopathological point of view and from the perspective of MR imaging, in two groups of patients with uveal melanoma: a group who had undergone previous proton beam radiotherapy (secondary enucleation); a control group who had undergone enucleation without any previous radiotherapy treatment (primary enucleation). Irradiated and nonirradiated uveal melanomas differ on the basis of the histological appearance, the MR imaging appearance and the distribution of necrosis. We hope that the findings we observed could be extended to all patients with uveal melanomas treated with radiotherapy, and may enhance the accuracy of radiologists in evaluating MR examinations after radiotherapy.

**Abstract:**

Necrosis in uveal melanomas can be spontaneous or induced by radiotherapy. The purpose of our study was to compare the histopathologic and MRI findings of radiation-induced necrosis of a group of proton beam-irradiated uveal melanomas with those of spontaneous necrosis of a control group of patients undergoing primary enucleation. 11 uveal melanomas who had undergone proton beam radiotherapy, MRI and secondary enucleation, and a control group of 15 untreated uveal melanomas who had undergone MRI and primary enucleation were retrospectively identified. Within the irradiated and nonirradiated group, 7 and 6 eyes with histological evidence of necrosis respectively, were furtherly selected for the final analysis; the appearance of necrosis was assessed at histopathologic examination and MRI. Irradiated melanomas showed a higher degree of necrosis as compared with nonirradiated tumors. Irradiated and nonirradiated lesions differed based on the appearance and distribution of necrosis. Irradiated tumors showed large necrotic foci, sharply demarcated from the viable neoplastic tissue; nonirradiated tumors demonstrated small, distinct foci of necrosis. Radiation-induced necrosis, more pigmented than surrounding viable tumor, displayed high signal intensity on T1-weighted and low signal intensity on T2-weighted images. The hemorrhagic/coagulative necrosis, more prevalent in nonirradiated tumors (4 out of 6 vs. 1 out of 7 cases), appeared hyperintense on T2-weighted and hypointense on T1-weighted images. Our study boosts the capability to recognize radiation-induced alterations in uveal melanomas at MRI and may improve the accuracy of radiologists in the evaluation of follow-up MR examination after radiotherapy.

## 1. Introduction

Uveal tract melanoma is considered a rare neoplasm, although it represents the most prevalent type of ocular melanoma and the most widespread primary intraocular malignancy among the adult population [1,2,3,4]. A variety of risk factors related to the onset of uveal melanoma have been identified: fair skin phototype, light iris, ultraviolet-B radiation exposure, melanocytic lesions both ocular or cutaneous, occupational cooking, welding [5,6,7].

From a histologic point of view, uveal melanoma can be pigmented, amelanotic or mixed [8,9]; the lesion is generally solid, though necrotic or hemorrhagic areas may occur as well. Necrosis, in particular, may produce cystic spaces or storage of eosinophilic debris within the neoplasm [10].

Almost one-third (30%) of patients with uveal melanoma is asymptomatic and the neoplasm is discovered during a routine eye examination; in the rest of the cases, the lesion can induce blurred vision, visual field defects, photopsia, metamorphopsia, and floaters. In case of tumor necrosis, pain, increased intraocular pressure and inflammatory changes of the sclera, episclera, and uvea may occur [5,11].

The diagnosis rests on ophthalmologic assessment, both in terms of clinical evaluation (slit-lamp biomicroscopy, indirect ophthalmoscopy) and imaging techniques (gonioscopy, ultrasonography, fundus fluorescein angiography, optical coherence tomography) [5]. Over the past few years, however, cross-sectional imaging methods have become more and more relevant in the diagnostic workup of uveal melanoma. Magnetic resonance imaging (MRI) provides crucial information about the local extent of the tumor affecting treatment planning; particularly, through a simultaneous 3D visualization of the tumor and organs-at-risk, MRI enables a more accurate and reproducible assessment of lesion dimensions than ophthalmic ultrasonography, thus shifting treatment strategy towards less invasive and expensive therapeutic options, in a considerable percentage (10-20%) of patients [12,13,14,15,16,17]. MRI, specifically diffusion-weighted imaging (DWI), provides equally important findings concerning tumor response to radiotherapy, useful also from a prognostic point of view [18,19]. Computed tomography (CT) and positron emission tomography/CT (PET/CT), owing to their comprehensive whole-body assessment, are useful in detecting distant metastases [6].

While in the past, invasive procedures were more often used to confirm or rule out the diagnosis of uveal melanoma, nowadays, in the cytogenetic age, tumor-sampling procedures (fine needle aspiration biopsy) have acquired an increasingly important prognostic role, making it possible to evaluate metastatic risk and to perform personalized treatments [20,21,22].

The vascular structure of the uvea explains the predominant hematogenous dissemination of the tumor (metastases occur in about 50% of patients), intrinsically linked to intratumoral angiogenesis [23]; on the other hand, regional lymphatic spread is exceedingly rare because of the paucity of the lymphatic system of the eye (limited to the conjunctiva and limbus) [6]. Uveal melanoma metastases demonstrate a particular tropism for the liver, representing the most frequent metastasizing site (93%), followed by the lungs (24%) and bones (16%) [6,24,25].

The purpose of the local therapy is to get local control of the neoplasm, to safeguard the eye and its visual function, and to prevent, or at least to minimize, the risk of metastatic spread [6,26]. In 1977 Zimmerman et al. reported an abrupt rise in the mortality rate of patients with uveal melanoma during the second year after enucleation (8%) as compared with the mortality rate before enucleation (1%). The authors hypothesized dissemination of tumor emboli during enucleation could be responsible for about 2/3 of the fatalities [27]. Since then, various eye-preserving radiotherapy techniques have been developed. Subsequent studies demonstrated similar survival rates between uveal melanoma patients treated with radiotherapy and those treated with enucleation [28,29,30], so that today eye-conserving irradiation has become the preferred treatment modality [31].

Although brachytherapy is the most widely employed among radiotherapy techniques for uveal melanomas, proton beam radiotherapy boasts a wider range of indications. The introduction of proton beam radiotherapy into clinical practice relies on the potential advantages related to its physical properties that allow: (1) to enhance the radiation dose towards the target to get better local tumor control and overall patient’s survival; (2) to cut down the dose to normal tissues adjacent to the target, thus averting radiation-induced damage or even radiation-induced secondary cancer [32].

Enucleation of the eyeball is currently performed in selected cases [33,34]. It is distinguished as: (a) primary when patients have not undergone any previous radiation eye-preserving therapy, and (b) secondary when patients have been formerly treated with laser techniques or radiation therapy [35].

When studying histologic changes attributable to irradiation, the comparison with a nonirradiated control group is very useful to recognize radiation-induced effects in the tumor. During the past decades, some studies have been carried out comparing an irradiated with a nonirradiated control group in order to differentiate the effects of radiotherapy from spontaneous degenerative changes, including necrosis, in uveal melanomas [36,37,38,39]; nevertheless, this topic has been investigated from the histopathological point of view, and a radiologic-pathologic correlation was not performed. Only in the last few years de Jong et al. and Ferreira et al. correlated MR findings with histopathology in retinoblastomas and uveal melanomas respectively [40,41]. However, in regard to both spontaneous and radiation-induced necrosis, some aspects are not yet fully understood, either in terms of pathogenetic mechanism or radiologic imaging.

In our study, we compared the histopathologic and MRI findings of radiation-induced necrosis of a group of proton beam-irradiated uveal melanomas (secondary enucleation) with those of spontaneous necrosis of a control group of patients that had undergone primary enucleation, in order to clarify the MR appearance of the two kinds of necrosis and the differences between them.

## 2. Materials and Methods

### 2.1. Study Population

The single-center, retrospective cohort study was approved by the institutional ethics review board of our institution (AOU “Policlinico-Vittorio Emanuele” Catania, Comitato Etico Catania 1, Protocol N. 24114, approved on 21 May 2021).

Patients were retrospectively identified from the database of the Anatomic Pathology Section and the search software of our picture archiving and communication system (PACS). All patients with a clinical diagnosis of uveal melanoma who had undergone proton beam radiotherapy at INFN LNS Nuclear Physics Laboratory, Catania between September 2016 and September 2021 were identified; among them, patients who thereafter underwent secondary enucleation were selected for potential inclusion in the study. The inclusion criteria were as follows: proton beam radiotherapy performed at INFN LNS Nuclear Physics Laboratory, Catania; secondary enucleation carried out within 2 weeks of an MR examination; MR examination performed at our department; final histologic diagnosis of uveal melanoma; presence of tumor necrosis at histological examination. The exclusion criteria were as follows: inadequate quality of MR images; incomplete MR protocol; radiotherapy different from proton beam; absence of tumor necrosis at histological examination.

As a control group, we selected a group of patients affected by uveal melanoma who had undergone primary enucleation without any previous radiotherapy treatment. The inclusion criteria for this comparison group of untreated melanomas were as follows: primary enucleation performed within 2 weeks of an MR examination; MR examination performed at our department; final histologic diagnosis of uveal melanoma; presence of tumor necrosis at histological examination. The exclusion criteria were as follows: inadequate quality of MR images; incomplete MR protocol; absence of tumor necrosis at histological examination.

All the patients granted informed consent concerning the use of their data for future scientific research.

### 2.2. Radiotherapy Protocol

**Figure 1:**
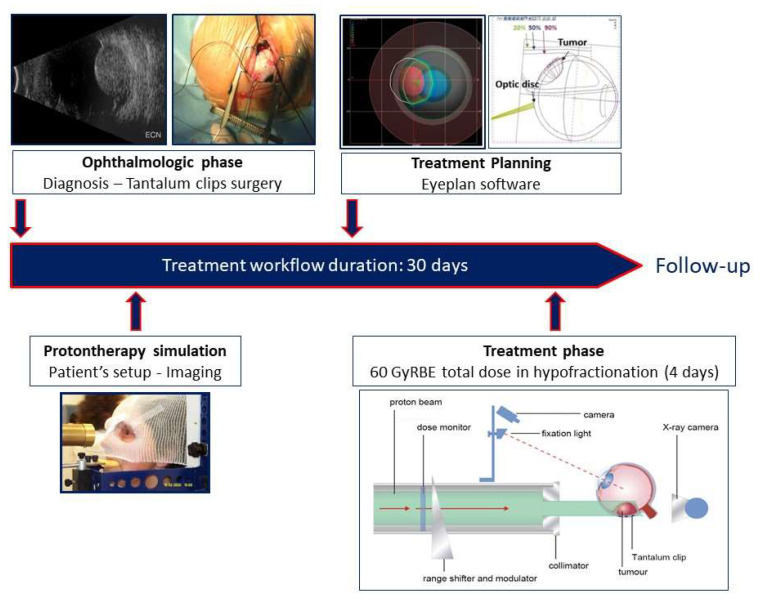
Proton beam radiotherapy workflow.

The treatment planning and the radiotherapy protocol have previously been described in detail [42,43] and are summarized in Figure 1.

### 2.3. MRI Protocol

MRI exams were conducted by means of a closed-configuration superconducting 1.5-T MRI scanner (Signa HDxT; GE Healthcare, Milwaukee, WI, USA) with 57.2 mT/m gradient strength and 120 T/m/s slew rate, through an 8-channel high-resolution neurovascular phased-array coil equipped with array spatial sensitivity technique (ASSET) parallel acquisition. All examinations included an MRI scan of the brain in addition to that of the orbit. Our MRI protocol has previously been described in detail [18] and is summarized in Table 1.

### 2.4. Histopathology

All enucleated eyes were fixed in 4% formaldehyde and paraffin processed; n. 8 sections passing through the pupil, the optic nerve, and the tumor were stained with hematoxylin-eosin as per routine protocol. All cases were jointly reviewed by two pathologists, R.C. (with 20 years of clinical experience in uveal melanomas) and G.B. (with 8 years of clinical experience in uveal melanomas); decisions were made by consensus. The pathologists were not blinded to the clinical and irradiation status of the patients. The tumors were classified according to the modified Callender classification [44].

The eyes of both the irradiated group (secondary enucleation) and the nonirradiated control group (primary enucleation) were examined for histopathologic evidence of necrosis. The degree of necrosis was evaluated in sections including the largest part of the neoplasm and was classified as follows: <10% (grade I), 10% to 50% (grade II), >50% (grade III).

### 2.5. Image Analysis

Histopathological slides and MR images were matched on the basis of well-defined anatomical landmarks (ciliary body, optic nerve head). MR images were reviewed by two radiologists, P.V.F. (with 10 years of experience in orbital imaging) and C.I. (with 4 years of experience in orbital imaging) in consensus. Here too, the radiologists were not blinded to the clinical and irradiation status of the patients. The MR appearance of tumor necrosis in both the irradiated and nonirradiated groups was evaluated on T1-weighted, T2-weighted, and diffusion-weighted imaging (DWI) acquisitions. As previously described by Ferreira et al., the signal intensity of necrotic areas on T1- and T2-weighted sequences was classified as hyper-, iso-, or hypointense, taking as a reference the signal intensity of the choroid and of the extrinsic eye muscles on T1- and T2-weighted images respectively [41]. In addition, the radiologists performed quantitative measurements of the apparent diffusion coefficient (ADC) of the tumors, as previously described [18], through a representative region of interest (ROI), taking care to exclude the most peripheral parts of the lesion, distortion artifacts, and macroscopically evident necrotic foci.

## 3. Results

### 3.1. Study Populaton

**Figure 2:**
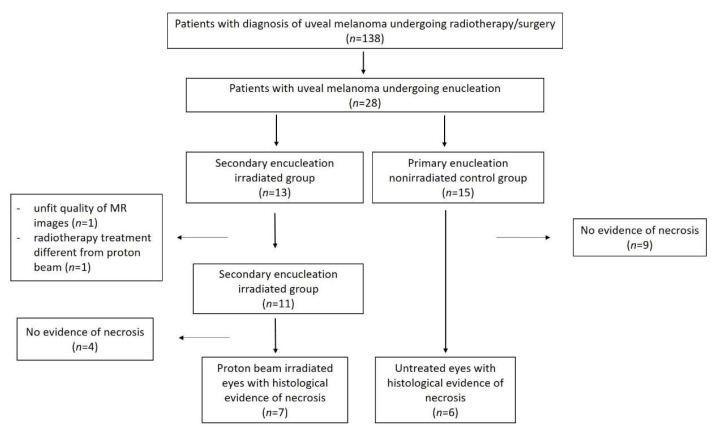
Flow diagram illustrating the patient selection process.

The patient selection process is summarized in Figure 2.

Based on the aforementioned inclusion and exclusion criteria, 13 patients of the irradiated group were selected for potential inclusion in the study. Of these patients, 2 were excluded because of the following reasons: unfit quality of MR images (*n* = 1), radiotherapy treatment different from proton beam (*n* = 1). Of the remaining 11 patients, 4 demonstrated no evidence of necrosis. Therefore, the enrolled patient cohort for the final histopathologic and MR imaging analysis consisted of 7 irradiated eyes with histological evidence of necrosis (irradiated group). The major reasons leading to enucleation in this irradiated study group were: clinically suspected tumor regrowth (6 patients) or severe radiotherapy-related complications (1 patient). MRI examinations were performed to more accurately assess the local extent of the tumor and to detect potential extrascleral extension (6 patients), and evaluate radiotherapy-related complications (1 patient). The time interval from proton beam radiotherapy to enucleation ranged between 18 and 46 months (average 33 months).

The control group of untreated melanomas (primary enucleation) consisted of 6 enucleated eyes with histological evidence of necrosis. These patients were selected out of a total amount of 15 patients with uveal melanoma who had undergone enucleation and had not received previous radiotherapy, during the time of this study. The demographic and clinicopathological characteristics of uveal melanoma patients are summarized in Table 2 and Table 3.

The patients included in our study are part of a wider group of 138 patients diagnosed with uveal melanoma during the period of the study.

### 3.2. Histopathologic Findings

The histopathological features of uveal melanomas in the irradiated and nonirradiated group are summarized in Table 4 and Table 5, respectively.

#### 3.2.1. Histopathologic Findings in the Irradiated Group

In the irradiated group, the mean tumor largest diameter was 6.1 mm (range, 1–14 mm); the mean tumor thickness was 4 mm (range, 0.2–8 mm). N. 2 tumors showed grade I necrosis; n. 1 case displayed grade II necrosis; n. 4 tumors exhibited grade III necrosis. The maximum diameter of necrotic areas ranged between 0.2 mm and 3.6 mm.

**Figure 3:**
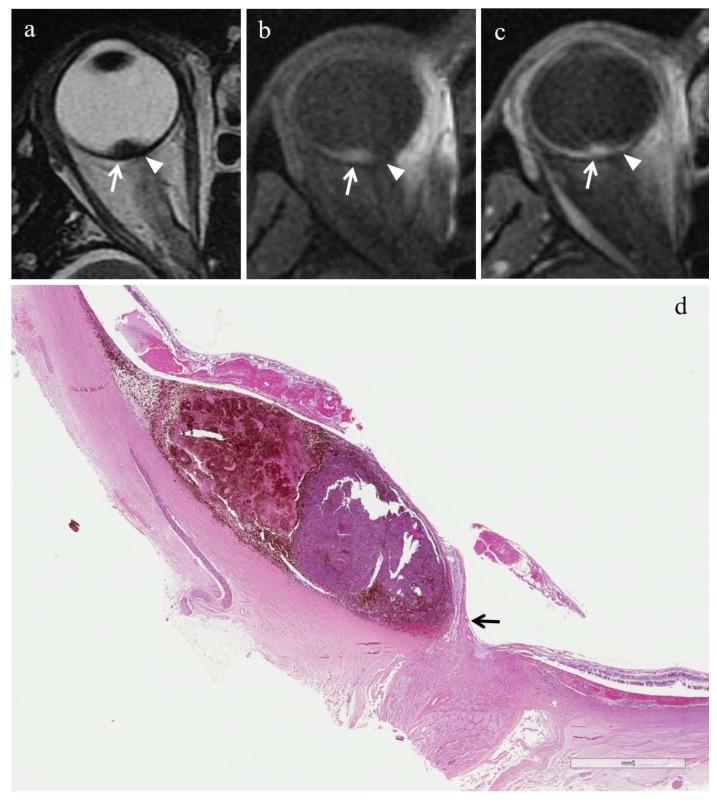
Radiation-induced necrosis. A 54-year-old male patient with a choroidal melanoma of the right eye. The patient underwent secondary enucleation 36 months after proton beam radiotherapy because of tumor regrowth. Axial (**a**) T2-weighted turbo spin-echo and (**b**) fat-suppressed T1-weighted mages demonstrate a lentiform-shaped intraocular lesion along the posterior aspect of the globe, adjacent to the optic nerve head. Two distinct portions are recognizable within the lesion. A lateral area (white arrows), hypointense on T2-weighted and hyperintense on T1-weighted images, representing radiotherapy-related necrosis; a medial portion (white arrowheads) with intermediate signal intensity, indicative of viable neoplastic tissue related to tumor recurrence of a poorly pigmented melanoma. On (**c**) axial contrast-enhanced fat-suppressed T1-weighted image the viable neoplastic tissue enhances (white arrowhead) and the distinction between the two portion of the lesion is hardly recognizable because of the intrinsic hyperintensity of radiotherapy-related necrosis (white arrow). (**d**) Histological low magnification (H&E, original magnification 50×) showing the conventional pattern of radiation-induced necrosis in uveal melanoma. A sharp demarcation between the residual poorly pigmented, viable tumor mass (on the right) and the abundant and heavily pigmented necrotic tissue (on the left) is seen. Note the emergence of the optic nerve (black arrow), compressed but not infiltrated by the tumor.

**Figure 4:**
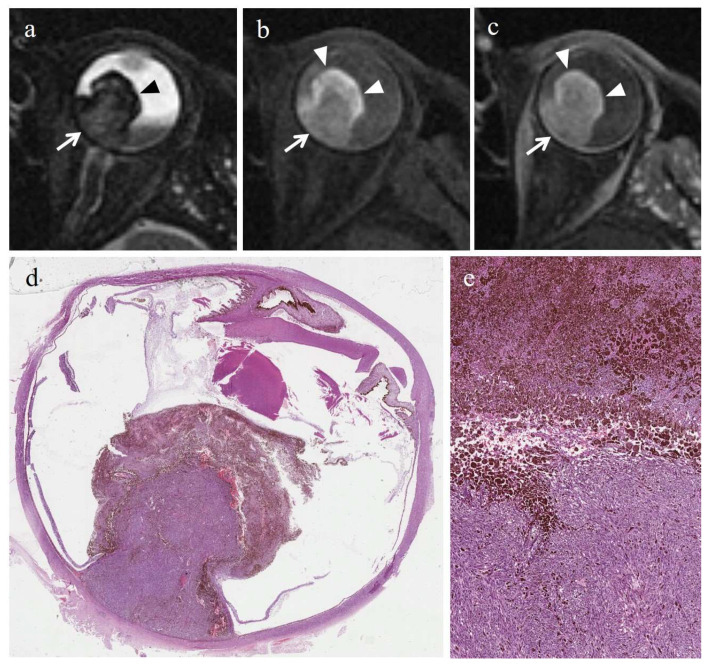
Radiation-induced necrosis. A 60-year-old female patient with a choroidal melanoma of the left eye. The patient underwent secondary enucleation 29 months after proton beam radiotherapy because of tumor regrowth. Axial (**a**) T2-weighted turbo spin-echo STIR and (**b**) fat-suppressed T1-weighted images show a mushroom-shaped intraocular lesion along the posterior aspect of the globe, covering the optic disc. Two different portions of the lesion are identifiable. A dorsal component (white arrows), adjacent to the posterior wall of the globe, with intermediate signal intensity, consistent with viable neoplastic tissue related to regrowth of a poorly pigmented melanoma; a ventral superficial portion (black arrowhead in a, white arrowheads in (**b**)), adjacent to the vitreous body, hypointense on T2-weighted and hyperintense on T1-weighted images, consistent with radiotherapy-related necrosis. On (**c**) axial contrast-enhanced fat-suppressed T1-weighted image the viable neoplastic tissue demonstrates enhancement (white arrow); the distinction between the two components of the mass is still recognizable, although is less evident as compared to unenhanced T1-weighted images, because of the intrinsic high signal intensity of radiotherapy-related necrosis (white arrowheads). (**d**) Low magnification (H&E; original magnification 25×) showing a poorly pigmented polypoid vital tumor mass surrounded by a well-defined and heavily pigmented necrotic “ring”. (**e**) Histological detail (H&E; original magnification 200×) illustrating the abrupt transition between the vital tumor (at the bottom) and radiation-induced necrosis (at the top).

**Figure 5:**
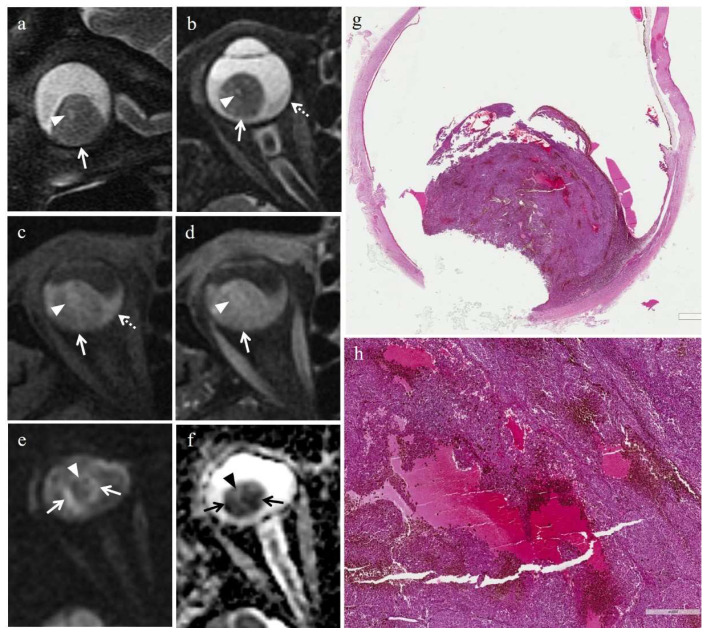
Hemorrhagic/coagulative-type necrosis in irradiated group (secondary enucleation). A 78-year-old female patient with a choroidal melanoma of the right eye. The patient underwent secondary enucleation 18 months after proton beam radiotherapy because of tumor regrowth. Sagittal (**a**) and axial (**b**) T2-weighted turbo spin-echo STIR images and (**c**) axial fat-suppressed T1-weighted mage display an intraocular dome-shaped mass along the postero-inferior aspect of globe (white arrows). An oval-shaped area, hyperintense on T2-weighted images and hypointense on T1-weighted image, surrounded by a thin peripheral rim, hypointense on T2-weighted and hyperintense on T1-weighted sequences (white arrowheads) is detectable within the lesion; the findings are consistent with hemorrhagic/coagulative-type necrosis. Note the hemorrhagic retinal detachment on both sides of the mass (white dotted arrow in (**b**) and (**c**)), better visible on T1-weighted sequences in which it appears hyperintense because of the shortening effect of subacute blood products (methemoglobin). On (**d**) axial contrast-enhanced fat-suppressed T1-weighted image the viable neoplastic tissue of the mass enhances (white arrow); the focus of hemorrhagic/coagulative-type necrosis, without enhancement, is still recognizable (white arrowhead). On (**e**) axial DW image (b = 1000 s/mm^2^) and (**f**) corresponding ADC map the viable neoplastic tissue shows restricted diffusion with high signal intensity on DWI image (white arrows) and low signal intensity on ADC map (black arrows); the necrotic focus does not exhibit restricted diffusion and is hypointense on DWI image (white arrowhead) and slightly hyperintense on ADC map (black arrowhead). (**g**) On low magnification (H&E, original magnification 25×), histological examination shows a largely viable and poorly pigmented residual tumor mass, located at the posterior segment of the eye, that contains multiple intratumoral foci of intratumoral hemorrhagic/coagulative-type necrosis. (**h**) Higher magnification (H&E, original magnification 150×). Although the case is from a secondary enucleation, necrotic foci exhibit abundant fibrin deposition, hemorrhagic infarction and are rimmed by heavily-pigmented extravasated melanophages. Note the striking consistency between the histologic and MR findings with reference to the necrotic focus.

In the majority of cases (6 out of 7) radiation-induced necrosis was characterized by a sharp demarcation from the residual viable tumor tissue and by a high degree of pigmentation (higher than that of surrounding viable tumor), due to an abundant infiltration of melanophages (Figure 3 and Figure 4). Multiple small foci of necrosis, exhibiting, as a peculiar feature, moderate hemorrhagic infarction, characterized by fibrin deposition and red blood cell extravasation, were found within viable tumor tissue only in 1 out of 7 cases (Figure 5) (Table 4).

#### 3.2.2. Histopathologic Findings in the Control Group

In the control group of untreated melanomas, the mean tumor diameter was 14.75 mm (range, 10.5–20 mm); the mean tumor thickness was 11.48 mm (range, 8–15 mm). N. 5 tumors showed grade I necrosis; n. 1 case exhibited grade II necrosis. The maximum diameter of necrotic areas ranged between 0.8 mm and 2 mm.

**Figure 6:**
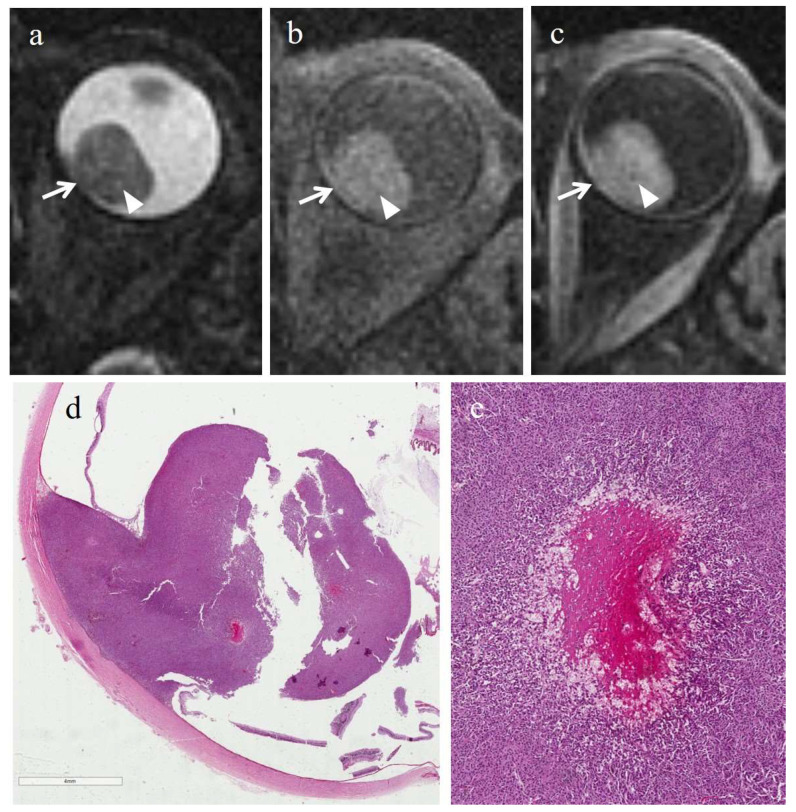
Hemorrhagic/coagulative-type necrosis in nonirradiated group (primary enucleation). A 79-year-old female patient with a poorly pigmented choroidal melanoma of the left eye. Axial (**a**) T2-weighted turbo spin-echo STIR, (**b**) fat-suppressed T1-weighted and (**c**) contrast-enhanced fat-suppressed T1-weighted images show a large intraocular collar-button mass along the posterior aspect of the globe (white arrows), demonstrating intermediate signal intensity on T2-weighted and plain T1-weighted images, and enhancement after contrast agent administration on T1-weighted image. Some small rounded areas, relatively hyperintense on T2-weighted image (white arrowhead in (**a**)), without enhancement on contrast-enhanced fat-suppressed T1-weighted mage (white arrowhead in (**c**)) are perceivable inside the lesion and hardly detectable on unenhanced T1-weighted image (white arrowhead in (**b**)). (**d**) Histologically, low magnification (H&E, original magnification 25×) illustrates a poorly pigmented, protruding melanoma mass of the posterior segment of the eye, that induces retinal detachment. (**e**) Histological detail (H&E, original magnification 150×) of a focus of intratumoral hemorrhagic/coagulative-type necrosis, characterized by fibrin deposition and hemorrhagic infarction, and rimed by extravasated histiocytes.

In 5/6 cases, tumor necrosis was present in the form of multiple, small necrotic foci interspersed among viable neoplastic cells; necrotic areas exhibited a high degree of pigmentation due to pigmented melanophages. Moreover, hemorrhagic-type necrosis, characterized by fibrin deposition and red blood cell extravasation, was found in 4 out of 6 cases (Figure 6). In 3/6 cases, the two kinds of necrosis coexisted (Table 5).

### 3.3. MRI Findings

The MRI features of uveal melanomas in the irradiated and nonirradiated group are summarized in Table 6.

#### 3.3.1. MRI Findings in the Irradiated Group

At MRI, necrosis was detectable in 6/7 irradiated eyes with histopathologic evidence of necrosis.

Radiation-induced necrosis displayed high signal intensity on T1-weighted images, low signal intensity on T2-weighted images, no evidence of restricted diffusion on DW sequences, and no evidence of enhancement on T1-weighted images acquired after contrast agent administration (Figure 3 and Figure 4). In cases where radiation-induced necrosis and viable tumor tissue coexisted, the boundary between them was very stark and well defined (Table 6). Radiation-induced necrosis was better visible on T2-weighted and on pre-contrast T1-weighted images than on post-contrast T1-weighted images, particularly in poorly pigmented melanomas (Figure 3 and Figure 4).

In the patient with histological evidence of hemorrhagic infarction, the hemorrhagic necrosis showed low signal intensity on T1-weighted images, high signal intensity on T2-weighted images, no restriction on DW images, and no evidence of enhancement on post-contrast T1-weighted sequences; moreover, the necrotic focus was surrounded by a thin peripheral rim, hyperintense on T1-weighted sequences and hypointense on T2-weighted sequences (Figure 5).

Three uveal melanomas of the irradiated group were excluded from ADC measurement, because of extensive necrosis with hardly recognizable tumor tissue or technical issues. In the irradiated group, the mean ADC (SD) of uveal melanomas was 0.78 × 10^−3^ mm^2^/s ± 0.08 (Table 7).

#### 3.3.2. MRI Findings in the Control Group

At MR imaging, necrosis was detectable only in 1/6 nonirradiated tumors with histopathologic evidence of necrosis. The patient in question exhibited hemorrhagic-type necrosis. In this case, the necrotic area demonstrated a cystic-like appearance, was relatively hypointense on T1-weighted images and hyperintense on T2-weighted images, compared with the surrounding tumor, and displayed no enhancement on T1-weighted images acquired after Gd-based contrast agent (Figure 6) (Table 8).

In the control group the mean ADC (SD) of uveal melanomas was 0.92 × 10^−3^ mm^2^/s ± 0.21.

In the remaining 1/7 patients of the irradiated group and 5/6 patients of the control group, the neoplasm exhibited just microscopic areas of necrosis, exclusively detectable at histopathologic examination, but below the spatial resolution of MR sequences.

## 4. Discussion

In the mid-80s, in one of the first reports dealing with pathologic features of uveal melanomas treated with proton beam radiotherapy, Ferry et al. postulated that it was not possible histopathologically to distinguish radiation-induced necrosis from spontaneous necrosis, since many untreated melanomas exhibit areas of spontaneous necrosis [45]; on the other hand, during the following years, various authors have demonstrated that eyes with secondary enucleation after radiotherapy displayed different histological features from eyes undergoing primary enucleation [38,39]. The common denominator of these reports is that tumors of the irradiated series are more likely to demonstrate signs of necrosis and a higher degree of necrosis than nonirradiated tumors; moreover, other degenerative changes, such as fibrosis, are more commonly encountered in irradiated tumors than in nonirradiated ones [36,38,39].

In our study, we focused our attention on pathologic findings, MR imaging findings, and MR imaging-pathologic correlation of treated uveal melanomas in order to understand how radiations modify tumor appearance at imaging, in particular with regard to radiation-induced necrosis.

The findings of our study are consistent with previous reports, since also in our study cohort irradiated tumors demonstrated a higher degree of necrosis as compared with the control group of primary enucleations, moreover, necrosis was histologically observed in 7/11 (64%) irradiated tumors and in 6/15 (40%) nonirradiated tumors. As one might expect, the tumors of the control group were bigger than those of the irradiated group, since in the former group, in most cases (4/6) the reason for enucleation was exactly a “large tumor”.

However, although radiotherapy has a noticeable effect on the viability of malignant cells and is able to produce considerable tumor regression, it rarely manages to destroy all neoplastic cells in uveal melanomas. In their review analyzing several reports published from 1977 to 1990, dealing with irradiated choroidal melanomas, Manschot et al. found that whilst tumor necrosis was present in 61% of microscopic examinations (a result very similar to our), viable tumor cells were still observable in 93% of cases [46]. The Collaborative Ocular Melanoma Study (COMS) subsequently confirmed that external beam radiotherapy significantly decreases, but does not eradicate mitotic activity [47]. Therefore, irradiation is not always able to destroy the reproductive capability of all malignant stem cells. Different reasons can explain the relative radioresistance of uveal melanomas: (1) the ability to repair sublethal radiation-induced damage because of a particularly efficient repair system; (2) a usually considerable amount of poorly oxygenated cells needing high radiation dose [46].

Furthermore, it should not be forgotten that radiation-induced necrosis is always the result of a multifactorial process, implying both direct (through chromosomal injury) and indirect effects. Radiotherapy may induce necrosis when neoplastic cells enter mitosis; therefore, cells apparently viable at standard light microscopic techniques may be programmed to die during the following mitotic cycle or may even pass numerous postradiation divisions before their metabolic death. Probably, for this reason, the tumors with a long intermitotic phase and slow turnover, including melanomas, may show no evidence or low degree of tumor necrosis and preserve metabolic integrity for a relatively long period after radiotherapy [36,38]. Therefore, discriminating viable from lethally damaged cells is not always possible at light microscopy. Aside from this direct cytotoxic effect, radiotherapy may indirectly induce cellular death through damage of neoplastic vasculature with consequent ischemia and through the host immune response against the irradiated neoplastic tissue [38,39,47,48].

Nevertheless, what we were most interested in was the histologic and MR appearance of tumor necrosis in irradiated and nonirradiated tumors.

The findings of our study suggested that the histological appearance and the distribution of necrosis tend to be different in irradiated versus nonirradiated tumors. In more detail, the former exhibited large and confluent necrotic foci, sharply demarcated from the residual viable neoplastic tissue, whereas nonirradiated lesions showed multiple small, distinct foci of necrosis within tumor tissue; this latter histologic pattern of necrosis was found in all nonirradiated tumors and only in 1 out of 7 irradiated lesions.

As mentioned above, in our study cohort, in the irradiated group the boundary between necrotic alterations and the adjacent viable tumor tissue was very well defined, both at histologic examination and at MR imaging, besides, necrotic areas were more pigmented than adjacent viable tumor tissue. The well-delimited boundaries of necrotic areas are due to the physical properties of charged particles. Proton beams, indeed, are collimated with extreme precision and deliver the bulk of their energy at a fixed depth, depending on the initial kinetic energy of the particles, thus focusing a noticeable radiation dose into a relatively limited volume of tissue [49].

We found that radiation-induced necrosis demonstrated a more pronounced pigmentation than that of surrounding viable tumor; this finding was related to an abundant infiltration of melanophages within necrotic areas. Precisely this copious presence of melanophages accounts for the MR appearance of radiation-induced necrosis and, in particular, for the high signal intensity on T1 weighted images and low signal intensity on T2-weighted images. Due to its imaging features, radiation-induced necrosis was better demonstrated on unenhanced T1-weighted images than on contrast-enhanced T1-weighted images, especially in poorly pigmented melanomas. Radiation-induced necrosis, characterized by very low signal intensity on T2- and high signal intensity on T1-weighted images, stands out clearly in the background of intermediate signal intensity of poorly pigmented melanomas. After the administration of the paramagnetic contrast agent, the viable tumor tissue enhanced, and as a result, on post-contrast T1-weighted images the intrinsic contrast between radiation-induced necrosis and adjacent neoplastic tissue decreased (Figure 3 and Figure 4).

In this respect, it should be emphasized the important contribution provided by the recent article of Ferreira et al. about the optimization of MR protocol in studying the eye and, in particular, uveal melanomas, in which the role—as well as the advantages and drawbacks of each pulse sequence—are admirably described. Although gradient-echo (GE) sequences have higher contrast resolution than spin-echo (SE) sequences and better depict the heterogeneity of the tumor (including necrosis), the authors removed GE sequences from the final clinical protocol because of their reduced capacity in discriminating the different layers of the globe [13]. In accordance with the abovementioned authors and taking into account the fact that we do not have a dedicated surface coil, our MR protocol includes only SE sequences.

One might wonder whether the degree of tumor pigmentation may affect the histologic and even the MR appearance of tumor necrosis. Actually, this relationship has not been proved yet and could be an interesting discussion topic for future researches.

In order to discriminate the effects of proton beam radiotherapy from spontaneous degenerative changes (in particular necrosis) in uveal melanomas, we have used a control group of nonirradiated melanomas that had undergone primary enucleation.

Spontaneous focal necrosis of uveal melanoma is indeed a quite common event [50], being reported in 5.7–33% of the cases in different series [38,39,51]; nevertheless, spontaneous massive tumor necrosis (degree of necrosis ≥ 80%) is rare, ranging between 0.5% and 3.6% [51,52]. In patients with uveal melanoma a wide range of symptoms and clinical signs can be associated with tumor necrosis: pain, poor vision, ocular and orbital inflammation, glaucoma, retinal detachment, vitreous hemorrhage, hyphema, exophthalmos [50,51,52,53,54,55,56,57]. In particular, according to Thareja et al., acute pain is the most common clinical finding, being present in all patients of their study [53]. Moreover, in some case reports and small case series, severe ocular pain was almost always associated with necrotic uveal melanomas [11,58].

The pathogenetic mechanism of spontaneous necrosis in uveal melanomas has not yet been completely elucidated. In early reports, some authors [50,59] postulated that the mass effect of the neoplasm would determine acute angle closure and secondary glaucoma, with a resultant increase in intraocular pressure above systemic blood pressure; this, in turn, leads to vascular compromise and ischemic necrosis of the melanoma and ocular tissues. More recently, other authors [52,53] focused their attention on vascular impairment as the *primum movens* initiating the necrotic process. In uveal melanomas characterized by particularly rapid growth, the vascular supply may become insufficient, leading to tumor necrosis. In particular, uveal melanomas located at the equatorial choroid (“watershed area”) would be particularly prone to infarction because of reasons related to their vascular anatomy. These authors have hypothesized that when uveal melanomas outgrow their own blood supply an ischemic necrosis occurs in the central portion of the tumor with release of proinflammatory cytokines resulting in perivascular inflammation, oedema and further necrosis. In this setting, anterior displacement of the iris diaphragm often occurs, with secondary angle-closure glaucoma and increase in intraocular pressure, furtherly enhancing tumor necrosis that may broaden involving also the iris and ciliary body [52,53]. Then again, the association of necrosis in uveal melanomas, with intraocular inflammation (scleritis, episcleritis, uveitis, and panophthalmitis), secondary angle-closure glaucoma, and increase in intraocular pressure has long been known [11,51,55,60]. Regardless of which of the two abovementioned hypotheses is the most reliable, the pathogenesis of spontaneous neoplastic necrosis seems to be a complex and multifactorial process.

In this regard, we would like to emphasize that the pattern of massive spontaneous necrosis described in the aforementioned previous reports [52,53], tends to be mainly a hemorrhagic/coagulative-type necrosis, that can be distinguished from the equally extensive radiation-induced necrosis, but not of hemorrhagic-type, typically seen in irradiated tumors.

However, in our study cohort, none of the cases of the comparison control group of untreated melanomas showed extensive necrosis >50%, and 5/6 (83%) patients had necrosis <5%. Our finding is consistent with that of previous authors. In the study of Crawford et al., none of the nonirradiated tumors demonstrated necrotic alterations >50%, and 27/30 (90%) of untreated melanomas displayed necrosis <5% [36]. Saornil et al. and Avery et al. found necrotic alterations >50% only in 4% and 1% of patients of the nonirradiated control group, respectively [38,39].

Saornil et al., in particular, first described a variant pattern of necrosis resembling coagulative necrosis (less pigmented than surrounding non-necrotic tumor) in both uveal melanomas treated with proton beam radiotherapy and in nonirradiated uveal melanomas (control group) [38]. Similarly, we found the presence of hemorrhagic/coagulative infarction, in both patients’ groups, however, conversely from the abovementioned authors, in our study cohort, the hemorrhagic/coagulative type necrosis was more commonly found in nonirradiated cases than in those irradiated (4 out of 6 vs. 1 out of 7 cases).

Moreover, this kind of hemorrhagic/coagulative type necrosis demonstrated a peculiar appearance at MR imaging. Necrosis appeared in the form of small areas, hyperintense on T2-weighted images, and hypointense on T1-weighted images, without enhancement after administration of contrast agent. Moreover, in the patient of the irradiated group, the hemorrhagic necrotic focus showed a thin peripheral rim, hypointense on T2-weighted, and hyperintense on T1-weighted sequences.

Interestingly, in 3/6 (50%) tumors of the nonirradiated group, we observed the simultaneous presence of the two kinds of necrosis. It is possible to hypothesize that tumor necrosis might represent the evolution of hemorrhagic/coagulative type necrosis; however, the small cohort of patients and the study design of our research do not allow to address this particular question. This appealing hypothesis, obviously hard to prove from a histological point of view, could instead be potentially confirmed in the future through periodic follow-up with MR examinations.

The mean ADC of ocular melanomas we measured in the present cohort, in both the irradiated and the control group (0.78 × 10^−3^ mm^2^/s and 0.92 × 10^−3^ mm^2^/s respectively) is lower than that reported in a recently published study by Ferreira et al. [41] but is only partially inconsistent with this latter. In our opinion such a low ADC value found in the control group could probably be related to our study cohort (large tumors needing enucleation); it is, in fact, closer to that reported by Erb-Eigner et al. [61] in whose study uveal melanomas had a mean diameter of 14 mm (very similar to our). In line with this, also Ferreira et al. found uveal melanomas with larger tumor prominence having lower ADC values [41]. The even lower ADC value we found in the irradiated group could be associated with tumor aggressiveness since these uveal melanomas represent tumor regrowth (recurrence), but honestly, the number of available measurements (only four) is too small to make any assumptions.

In previous studies, we investigated the role of DWI sequences in predicting and detecting the response of ocular melanoma to proton beam radiotherapy [18,19]. Necrosis affects MR functional quantitative parameters, such as ADC; we know in fact that induction of necrosis by cytotoxic treatment determines an increase in tumor ADC detectable prior to changes in tumor volume. When measuring ADC value on DWI sequences, including microscopic necrotic foci in the ROI is unavoidable, nevertheless recognizing macroscopic necrotic areas and excluding the latter from ADC measure may allow for a more accurate and reliable assessment. For this reason, we believe that it might be important, to recognize radiation-induced necrosis and we think that MRI can still be used as an early marker of therapy response.

Undoubtedly, some limitations of our preliminary study need to be acknowledged. The major limitation is the small cohort of patients. Because of the small sample size, in our study irradiated and nonirradiated tumors were not matched on the basis of clinical prognostic factors (i.e., metastatic risk) and therefore, differences between groups would be attributable, at least to some extent, also to different baseline features of the tumors. An important limitation, representing a potential source of bias, is the lack of pre-treatment MR examinations in the irradiated group. This prevented us from knowing for sure whether necrotic foci were already present before the proton beam radiotherapy. Nevertheless, the fact that we found a higher degree of necrosis in the irradiated group than in the nonirradiated control group, although tumors of this latter were much bigger, let us hypothesize that necrotic areas we found in patients of the irradiated group were mostly attributable to the effect of radiotherapy. The fact that neither the radiologists nor pathologists were blinded to the clinical and irradiation status of the patients could apparently lead to bias, too. Nevertheless, we thought this was the best choice for our study since the aim of our work was not to assess the capability of MRI to distinguish spontaneous from radiation-induced necrosis, but rather to compare the histopathologic and MRI findings of radiation-induced and spontaneous necrosis in order to clarify the histologic and MR appearance of the two kinds of necrosis in uveal melanomas. Indeed, the retrospective study design is a limitation, however, given the rarity of the disease and of the enucleation procedure, a retrospective approach was probably the most feasible and accomplishable study design. Another limitation is the heterogeneity of our study cohort and in particular the high percentage of patients with grade I necrosis in the control group; indeed, in most of these patients the necrotic alterations were not appreciable at MR imaging. Nevertheless, the inability of MRI to detect microscopic necrotic foci is not surprising; previous authors have demonstrated that small morphological characteristics of retinoblastoma (including necrosis) were not appreciable through currently employed clinical in vivo 1.5 T MRI scanners, but only on ultra-high-field MRI (9.4 T and 17.6 T) [40]. Moreover, as mentioned above, a low degree of necrosis in the nonirradiated group is common also in other previous studies. Lastly, the limitations, both on the hardware and software side, related to our MRI apparatus and MRI protocol, should not be forgotten. We performed MR examinations through a 1.5 T scanner and a neurovascular array coil. The use and availability of MRI systems operating at higher magnetic field strengths (≥3 T) coupled with dedicated receive surface coils undoubtedly ensure higher spatial resolution and signal-to-noise ratio [13,41,62,63]. Moreover, although our MR protocol included DWI sequences, it lacks other functional sequences, such as dynamic contrast-enhanced (DCE)-MRI and perfusion weighted-imaging (PWI) that have proven to be useful in the differential diagnosis of intraocular tumors and in evaluating tumor response to radiotherapy and could have a promising value in detecting lesions with a more severe prognosis [13,41,63,64].

## 5. Conclusions

In our study, irradiated and nonirradiated tumors differed on the basis of the histological appearance, but above all on the basis of the distribution of necrosis. Irradiated tumors (secondary enucleation) showed large and confluent necrotic foci, sharply demarcated from the residual viable neoplastic tissue; on the other hand, in nonirradiated tumors (primary enucleation), necrosis, usually, took on the appearance of multiple small, distinct foci. Radiation-induced necrosis, more pigmented than surrounding viable tumor, had a peculiar appearance at MRI, related to the abundant presence of melanophages, showing high signal intensity on T1-weighted images and low signal intensity on T2-weighted images. The hemorrhagic/coagulative necrosis, more prevalent in nonirradiated tumors, appeared in the form of small areas, hyperintense on T2-weighted images, and hypointense on T1-weighted images.

Some questions remain unaddressed and could be the topic for future studies: (a) whether the degree of tumor pigmentation may affect the histologic and the MR appearance of tumor necrosis; (b) whether tumor necrosis and hemorrhagic/coagulative necrosis may represent each other’s evolution.

Despite the abovementioned limitations, our study, as well as confirming previous findings, contributes to our understanding of the effects of radiation on uveal melanomas and to our capability to recognize radiation-induced alterations at MRI. The importance of recognizing radiation-induced necrosis is twofold; recognizing necrosis allows us to evaluate the effects of radiotherapy and, at the same time, enables more accurate measurements of functional quantitative parameters (ADC value, perfusion characteristics) excluding potentially necrotic parts. Our hope is that the findings we observed could represent useful additional information when studying patients with uveal melanomas treated with proton beam radiotherapy, in order to enhance the accuracy of radiologists in the evaluation of follow-up MR examination after radiotherapy.

## Figures and Tables

**Table 1:** MRI protocol. Table sets out an overview of parameters of MRI pulse sequences. Fat-suppressed T1-weighted sequences were performed before and after i.v. administration of paramagnetic Gd-based contrast agent (0.2 mL per kilogram of body weight).

MRI Protocol	T2W FSE	T2W FSE STIR	T1W FSE	T1W FSE Fat Sat	DWI SE EPI
Acquisition plane	axial, coronal	axial, coronal	axial, coronal	axial, coronal	axial
Repetition time/echo time (msec)	3220/120	3700/50	550/14.9	450/15.1	4800/89.9
Flip angle	90°	90°	90°	90°	90°
Echo train length	19	12	2	2	-
N. of averages	4	3	3	2	8
Section thickness (mm)	3	3	3	3	4
Interslice gap (mm)	0.3	0.3	0.3	0.3	0.4
Field of view (mm)	160 × 160	160 × 160	160 × 160	160 × 160	200 × 200
Matrix	352 × 256	256 × 256	256 × 224	256 × 256	192 × 192
Frequency direction	Superior to inferior	Anterior to posterior	Right to left	Right to left	Right to left
b-value (s/mm^2^)	-	-	-	-	0–1000

T1W = T1-weighted, T2W = T2-weighted, FSE = fast spin-echo, STIR = short tau inversion recovery, fat sat = fat saturation (frequency-selective fat saturation), DWI = diffusion-weighted imaging, SE = spin-echo, EPI = echoplanar imaging.

**Table 2:** Demographic and clinicopathological characteristics of the irradiated group.

Patient	Gender	Age	Eye	Tumor Location	Interval between Irradiation and Enucleation	Reasons for Enucleation	Pretreatment Tumor Size * (TP, LBD)
1	Female	72	Left	Choroid	46 months	Radiotherapy-related complications	7.8 mm, 18 mm
2	Male	60	Right	Choroid	35 months	Tumor regrowth	6 mm, 13.4 mm
3	Male	29	Right	Choroid	34 months	Tumor regrowth	8 mm, 16.2 mm
4	Male	44	Right	Choroid	32 months	Tumor regrowth	2.7 mm, 8 mm
5	Female	78	Right	Choroid	18 months	Tumor regrowth	5.4 mm, 12.6 mm
6	Male	54	Right	Choroid	36 months	Tumor regrowth	4.2 mm, 9.1 mm
7	Female	60	Left	Choroid	29 months	Tumor regrowth	6.3 mm, 11.2 mm

TP: tumor prominence; LBD: largest basal diameter; * measured with ultrasonography.

**Table 3:** Demographic and clinicopathological characteristics of the nonirradiated group.

Patient	Gender	Age	Eye	Tumor Location	Reasons for Enucleation	Pretreatment Tumor Size * (TP, LBD)
1	Male	55	Left	Choroid	Optic nerve invasion	8 mm, 16 mm
2	Female	80	Right	Choroid	Large tumor	8 mm, 13 mm
3	Male	77	Right	Choroid	Optic nerve invasion	15 mm, 20 mm
4	Male	70	Right	Choroid	Large tumor	12.2 mm, 10.7 mm
5	Male	40	Left	Choroid	Large tumor	11 mm, 14 mm
6	Female	79	Left	Choroid	Large tumor	12.1 mm, 12.3 mm

TP: tumor prominence; LBD: largest basal diameter; * measured with ultrasonography.

**Table 4:** Histopathological features of uveal melanomas in the irradiated group.

Patient	Histologic Type	Degree of Pigmentation	Degree of Necrosis	Necrotic Pattern
1	Necrosis without viable tumor tissue	-	Grade III	Sharply demarcated tumor necrosis
2	Spindle cell	Pigmented	Grade I	Sharply demarcated tumor necrosis
3	Epithelioid cell	Pigmented	Grade II	Sharply demarcated tumor necrosis
4	Spindle cell	Pigmented	Grade III	Sharply demarcated tumor necrosis
5	Mixed cell type	Poorly pigmented	Grade I	Multiple foci- hemorrhagic/coagulative-type
6	Mixed cell type	Poorly pigmented	Grade III	Sharply demarcated tumor necrosis
7	Spindle cell	Poorly pigmented	Grade III	Sharply demarcated tumor necrosis

**Table 5:** Histopathological features of uveal melanomas in the nonirradiated group.

Patient	Histologic Type	Degree of Pigmentation	Degree of Necrosis	Necrotic Pattern
1	Epithelioid cell	Pigmented	Grade I	Multiple foci- hemorrhagic/coagulative-type, tumor necrosis
2	Epithelioid cell	Poorly pigmented	Grade I	Multiple foci- hemorrhagic/coagulative-type, tumor necrosis
3	Spindle cell	Pigmented	Grade II	Multiple foci tumor necrosis
4	Mixed cell type	Pigmented	Grade I	Multiple foci tumor necrosis
5	Spindle cell	Pigmented	Grade I	Multiple foci- hemorrhagic/coagulative-type, tumor necrosis
6	Mixed cell type	Poorly pigmented	Grade I	Multiple foci- hemorrhagic/coagulative-type

**Table 6:** Chart summarizing the MR imaging patterns of necrosis.

MR Finding	T2	T1	Gd-T1	DWI
Radiation-induced necrosis	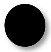 Low signal	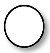 High signal	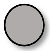 No enhancement	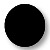 Low signal
Radiation-induced necrosis with viable tumor tissue	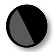 M RIN	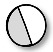 M RIN	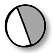 M RIN	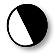 M RIN
Hemorrhagic necrosis in untreated melanoma	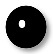 High signal	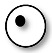 Low signal	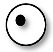 No enhancement	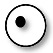 Low signal

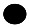
 low signal; 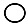 high signal; 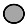 no enhancement; M: melanoma; RIN: radiation induced necrosis.

**Table 7:** MR imaging appearance * of necrosis of uveal melanomas in the irradiated group.

Patient	T2	T1	Gd-T1	DWI	ADC × 10^−3^ mm^2^/s **
1	Hypointense	Hyperintense	No enhancement	No restriction	-
2	-	-	-	-	0.76
3	Hypointense	Hyperintense	No enhancement	No restriction	0.84
4	Hypointense	Hyperintense	No enhancement	No restriction	-
5	Hyperintense	Hypointense	No enhancement	No restriction	0.67
6	Hypointense	Hyperintense	No enhancement	No restriction	-
7	Hypointense	Hyperintense	No enhancement	No restriction	0.86

* Reference: signal intensity of choroid on T1-WI and eye muscles on T2-WI. ** ADC value measured in the viable tumor tissue.

**Table 8:** MR imaging appearance * of necrosis of uveal melanomas in the nonirradiated group.

Patient	T2	T1	Gd-T1	DWI	ADC × 10^−3^ mm^2^/s **
1	-	-	-	-	1.04
2	-	-	-	-	1.22
3	-	-	-	-	0.63
4	-	-	-	-	0.78
5	-	-	-	-	1.05
6	Hyperintense	Hypointense	No enhancement	No restriction	0.80

* Reference: signal intensity of choroid on T1-WI and eye muscles on T2-WI. ** ADC value was measured in the viable tumor tissue.

## Data Availability

The data presented in this study are available on request from the corresponding author.
